# Exploring Deleterious Nonsynonymous SNPs in the *ACADM* Gene: Insights Into Medium‐Chain Acyl‐CoA Dehydrogenase Deficiency (MCADD) via In Silico Analysis

**DOI:** 10.1155/genr/6682668

**Published:** 2026-02-27

**Authors:** Muhammad Waleed Iqbal, Muhammad Shahab, Fakhreldeen Dabiellil, Yousef A. Bin Jardan, Mohammed Bourhia, Xinxiao Sun, Qipeng Yuan

**Affiliations:** ^1^ State Key Laboratory of Chemical Resources Engineering, Beijing University of Chemical Technology, Beijing, 100029, China, buct.edu.cn; ^2^ University of Bahr El Ghazal, Freedom Street, Wau, 91113, South Sudan; ^3^ Department of Pharmaceutics, College of Pharmacy, King Saud University, P.O. Box 11451, Riyadh, Saudi Arabia, ksu.edu.sa; ^4^ Laboratory of Biotechnology and Natural Resources Valorization, Faculty of Sciences, Ibn Zohr University, Agadir, 80060, Morocco, uiz.ac.ma

**Keywords:** *ACADM*, MCADD, MD simulation, mutational analysis, nsSNPs

## Abstract

Medium‐chain acyl‐CoA dehydrogenase deficiency (MCADD), a potentially lethal metabolic disorder, is often associated with single‐nucleotide polymorphisms (SNPs) in the acyl‐CoA dehydrogenase, medium‐chain (*ACADM*) gene. The current research investigates the structural and functional implications of these genetic variants through diverse bioinformatics techniques. A range of in silico techniques were utilized to thoroughly evaluate the effect of nonsynonymous SNPs. Molecular docking and molecular dynamics simulation evaluation comprehensively validated the mutational impact on protein’s stability. Gene interaction analysis demonstrated that *ACADM* is involved in several cellular pathways and co‐expression networks. Two nsSNPs, rs121434282 and rs200724875, were found to have a significant impact on the composition and functionality of *ACADM*. This research lays the foundation for precision medicine advancements, specifically against metabolic disorders. Thorough validation of the proposed nsSNPs, supported by animal models, is crucial for understanding their role in MCADD.

## 1. Introduction

The predominant type of genetic variation in humans is single‐nucleotide polymorphisms (SNPs), which are defined by a single‐nucleotide alteration at a specific genomic locus among distinct individuals. The coding sections of genes, which are in charge of converting genetic information into protein sequences, are where these alterations are most noticeable [1]. Among the most important SNPs are nonsynonymous SNPs (nsSNPs), which alter DNA sequence directly, and consequently protein’s amino acid sequence. These alterations can profoundly influence the structure and function of proteins, hence modifying an individual’s susceptibility to disease and overall health status. Interestingly, nsSNPs are confined to the coding area of a gene [2].

The diversity of human genome is largely defined by genomic variations, or nsSNPs, which can also significantly affect a person’s susceptibility to certain diseases by causing functional alterations in the genome [3]. Human genome variants have been widely associated with a variety of inherited metabolic disorders [4, 5]. One such condition is medium‐chain acyl‐CoA dehydrogenase deficiency (MCADD), a disorder caused by pathogenic mutations in the *ACADM* gene on chromosome 1p31. This gene encodes the MCAD enzyme, a crucial catalyst in the β‐oxidation of fatty acids, which is a critical pathway for energy synthesis under fasting or metabolic stress [6, 7]. Loss‐of‐function mutations in ACADM disrupt this process, leading to decreased fatty acid oxidation and resultant energy shortage, especially during fasting or illness. Because MCADD follows an autosomal recessive inheritance pattern, individuals must inherit pathogenic variants in both alleles of ACADM for clinical symptoms to manifest [8]. The disorder predominantly affects individuals of European descent, with a reported incidence ranging from 1 in 8,100 to 1 in 27,000 births [9]. Clinically, MCADD is characterized by episodes of hypoketotic hypoglycemia, vomiting, lethargy, seizures, and, in severe cases, coma or sudden death, typically triggered by prolonged fasting or infection [10]. Newborns may appear healthy at birth but can rapidly decompensate under catabolic stress when fatty acid oxidation becomes critical for energy supply. The phenotypic variability of MCADD arises largely from the nature of specific ACADM mutations, which determine the residual activity of the MCAD enzyme [11]. Some variants result in mild or late‐onset forms, while others cause severe metabolic crises. Even with early diagnosis and dietary management, affected individuals may experience long‐term complications such as hepatic dysfunction, muscle weakness, or neurodevelopmental delay [12]. These observations underscore the crucial role of the ACADM gene in maintaining metabolic homeostasis and the clinical importance of understanding how individual genetic variants alter MCAD enzyme structure and function.

In order to predict and understand their probable implications on protein function, we perform an in silico investigation of nsSNPs in the *ACADM* gene using computational techniques. Our objective is to uncover the molecular consequences of these genetic alterations, therefore offering important insights into the intricate relationship between metabolic pathways and genetic diversity. This work provides a framework for understanding how variations in genes lead to individual variability in disease risk and treatment responses, which has implications for personalized medicine in addition to improving our understanding of *ACADM* gene function. Additionally, this study will shed light on how certain mutations impact protein function and may contribute to a range of illnesses linked to the *ACADM* gene.

## 2. Methodology

**Figure 1:**
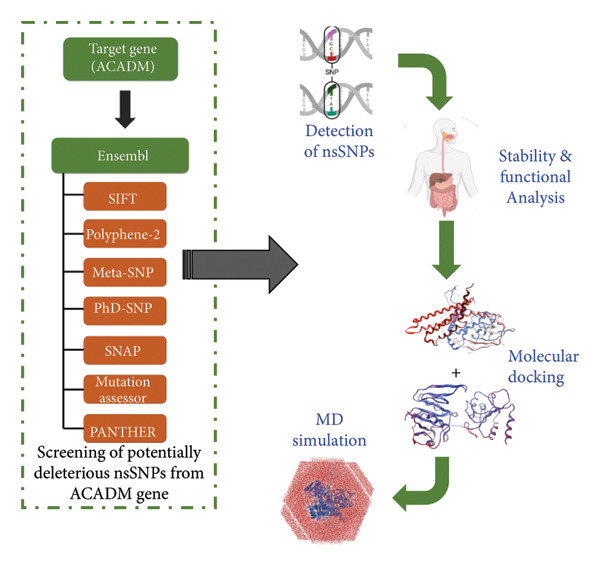
Workflow to identify the potentially damaging nsSNPs in ACADM gene.

A wide variety of distinguished approaches were utilized to perform mutational investigation, and Figure 1 displays the entire process. During execution, GRCh38 served as the reference human genome for all tools and web servers [13].

### 2.1. Acquisition of nsSNPs

All SNPs within the *ACADM* gene have been fully documented in the NCBI dbSNP database (https://www.ncbi.nlm.nih.gov/snp/), which also provides information on residual changes, locations, and worldwide minor allele frequencies (MAFs) [14]. 17,135 SNPs were discovered after the data were evaluated, 446 of which were nsSNPs. A consensus method was used to find an additional 488 missense SNPs using the Ensembl database (https://www.ensembl.org/) [15]. Extensive experiments were conducted to reduce duplication and improve the nsSNP list, ensuring accuracy.

### 2.2. Scanning of the Most Deleterious nsSNPs

To examine the potential relevance of acquired nsSNPs, we applied seven bioinformatics screening tools. These include the SIFT (Sorting Intolerant from Tolerant) (https://sift.bii.a-star.edu.sg/) [16, 17], PolyPhen‐2 (http://genetics.bwh.harvard.edu/pph2/) [18], Meta‐SNP (https://snps.biofold.org/meta-snp/) [19], PhD‐SNP (https://snps.biofold.org/phd-snp/phd-snp.html) [20], PANTHER (https://www.pantherdb.org/) [21], SNAP (http://snap.genomics.org.cn/) [22], and MutationAssessor (http://mutationassessor.org/r3/) [23] were employed. Finding nsSNPs that were constantly judged as detrimental or dangerous by all seven techniques was the goal. This all‐inclusive approach improves the precision of identifying nsSNPs and their likely functional implications.

### 2.3. Determination of the Influence on Structure and Function

After identifying probable detrimental nsSNPs, we examined their repercussions on the protein’s form and function. Herein, we applied the MutPred 1.2 (http://mutpred.mutdb.org/) [24] to explore the possible consequences of residual variations on protein structure. This tool examines the expected implications of mutations, such as alterations in phosphorylation sites and helical orientations. Using MutPred, we found harmful nsSNPs by inputting the *ACADM* protein sequence. Mutations were grouped based on their p values, with those less than 0.05 indicating average certainty and those under 0.01 expressing promising confidence.

### 2.4. *ACADM*’s Stability Assessment

Using the I‐Mutant 2.0 (https://folding.biofold.org/i-mutant/i-mutant2.0.html), we looked into the possible impacts of harmful nucleotide substitutions that were found on the *ACADM* protein stability [25]. This program estimates fluctuations in protein integrity brought on by variants via machine learning. I‐Mutant 2.0, which replicates the protein under normal physiological conditions (pH 7.0 and 25°C), was used to analyze the nsSNPs. A “reliability index” (RI) within 0 to 10 is calculated by this software, where higher numbers suggest better stability. Finding nsSNPs that would cause the *ACADM* protein to become unstable was the aim of analyzing these RI scores.

### 2.5. Conservatory Analysis of *ACADM*

Each of the protein residues was investigated for evolutionary conservation via DeepREx‐WS (https://deeprex.biocomp.unibo.it/) [26]. This online application assesses protein sequences and makes several predictions about them, including conservation, using deep learning. In order to examine protein sequences, the study makes use of deep neural networks, a kind of artificial intelligence that is modeled after the composition and operations of the human brain.

### 2.6. Three‐Dimensional (3D) Structure Prediction

We used a comprehensive method known as Robetta modeling (https://robetta.bakerlab.org/) [27] to study the likely repercussions of the most significant mutations on the 3D structure of the *ACADM* protein. Using the Rosetta program, this tool was applied to create 3D models of eight mutant proteins, along with the wild type, that had the most harmful nsSNPs. We worked with TM‐align (https://zhanggroup.org/TM-align/) to assess the 3D conformation of each mutant model with the protein’s wild‐type substitute. Data on morphological interpolation, the TM score, and root mean square deviation (RMSD) were obtained from this experiment. RMSD is used to calculate the standard deviation between the locations of matching atoms in two setups. More structural divergence is indicated by higher RMSD values [28]. The TM score, on the other hand, is a mathematical value between 0 and 1; 1 represents the maximum degree of structural homology. Based on the early investigation, the two variants with the greater morphological deviations (greater RMSD) from the wild type were chosen. The state‐of‐the‐art protein structure prediction tool AlphaFold2 (https://alphafold.ebi.ac.uk/) was then used to examine these [29]. The produced protein structures were interactively displayed using PyMOL software, allowing for a thorough examination of crucial characteristics [30].

### 2.7. Docking Analysis

*ACADM* mediates the dehydrogenation of medium‐chain acyl‐CoA molecules, transferring electrons to ETF (electron transfer flavoprotein), which then facilitates their entry into the respiratory chain. The formation of a complex between *ACADM* and ETF is essential for efficient electron transfer [6, 12, 31]. Disruptions in this interaction due to mutations can lead to MCADD. We applied computational docking approach to provide more clarity into the potential interconnection of wild‐type *ACADM* and the mutants with ETF protein. For this aim, we acquired 3D coordinates of ETF from Protein Data Bank (https://www.rcsb.org/) (PDB ID: 1EFV) [32]. Polar hydrogen atoms were introduced to the molecular framework, and the energy was minimized using the Molecular Operation System (MOE) software as a predocking step [33]. Next, we employed the powerful online tool ClusPro v2.0 docking server (https://cluspro.bu.edu/), which estimates protein–protein contacts via energy calculations [34]. By simulating potential interactions between the ETF protein and *ACADM* mutants, we were able to precisely dock them. Finally, we employed the PDBsum server (https://www.ebi.ac.uk/thornton-srv/databases/pdbsum/) to examine the details of these intermolecular contacts [35]. Via precisely defined residues, bonds, and forces, we are more capable to perceive the various paths that interact between *ACADM* and ETF.

### 2.8. Molecular Dynamics (MD) Simulation Analysis

MD simulations are commonly utilized to estimate atomic motion and protein–protein complex stability [36]. MD simulations were carried out simultaneously between the *ACADM*–ETF complex, along with the *ACADM* mutant–ETF complex (R206H, R281T). Free energies were calculated via the ff19SB force field within the Amber22 package to evaluate their effectiveness [37]. To begin, preparation built‐in program Tleap was implemented for building and handling the problems. Each system was neutralized using either Na^+^ or Cl^−^ complimentary ions [38]. To lower the energy and to resolve problematic conflicts within each balanced framework, we employed conjugate gradient, and the steepest descent as the two‐section energy optimization system [39]. We employed protein restrictions in the first phase to fix the optimized energy condition for water molecules in 2500 steps. This integrates 1000 steps for the steepest descent and 1500 steps for the conjugate gradient. During the subsequent phase, we removed all restraints and optimized the whole complex’ energy to 2500 steps (with the same step configurations). Then, the optimized complexes underwent heating for 50 ps at 300 K. A Berendsen barostat [40] was utilized to monitor the system pressure, and a Langevin thermostat [41] was used to control the temperature. The SHAKE algorithm was used to strengthen the covalent bonds [42]. After 1000 ps of equilibration, an NPT ensemble was used to compress the complex system [43]. The GPU version of Amber22 (PMEMD.cuda) was used to run MD simulations on four complexes [44]. Each compound underwent a 200‐ns simulation throughout the development phase, following the utilization of PTRAJ and CPPTRAJ to examine the obtained trajectories [45, 46].

### 2.9. Assessment of Post‐Translational Modification (PTM) Sites

We studied a spectrum of PTMs, reported to alter the functionality of proteins, gaining an improved comprehension of the mutational consequences. Utilizing GPS‐MSP (https://msp.biocuckoo.org/), putative methylation regions within the *ACADM* protein were found. The NetPhos 3.1 (https://services.healthtech.dtu.dk/services/NetPhos-3.1/) and the GPS 6.0 (https://gps.biocuckoo.org/online.php) were acquired to find the phosphorylation regions for particular residues such as tyrosine, threonine, and serine. Predictions using GPS 6.0 were thought to be greater in precision and dependability [47]. The Neural network clusters with a set criterion of 0.5 were used within NetPhos 3.1 [48]. Amino acids phosphorylated were those that scored higher than this threshold. Putative ubiquitination regions were discovered via the RUBI (https://old.protein.bio.unipd.it/rubi/) and the GPS‐Uber (https://gpsuber.biocuckoo.cn/) utilities, where lysine received special attention. RUBI used an equitable criterion to account for the possibility of ubiquitination for lysine residues [49]. NetOglyc4.0 (https://services.healthtech.dtu.dk/services/NetOGlyc-4.0) was applied to determine glycosylation sites within the *ACADM* protein [50]. Using this technique, the predicted glycosylation trends of the mutants and wild type were compared in order to identify potential functional differences caused by mutations.

### 2.10. *ACADM* Gene–Gene Interaction

GeneMANIA (https://genemania.org/) [51] and STRING (https://string-db.org/) [52] were utilized to investigate the changes in the *ACADM* protein and its relationships with other genes caused by the identified nsSNPs. To analyze the gene–gene connections, GeneMANIA uses diverse sources, for example, physical interactions, shared routes, and co‐expression. It provides an intricate map showing possible intergene associations, while the STRING emphasizes on interprotein connections and finds the most important *ACADM*‐interacting genes by utilizing diverse sort of data, including clinical evidence, co‐expression, and co‐occurrence. Greater relationships are reflected in higher scores. A scale from zero to one is used to rate the encounters.

## 3. Results

### 3.1. Retrieved nsSNPs

**Figure 2:**
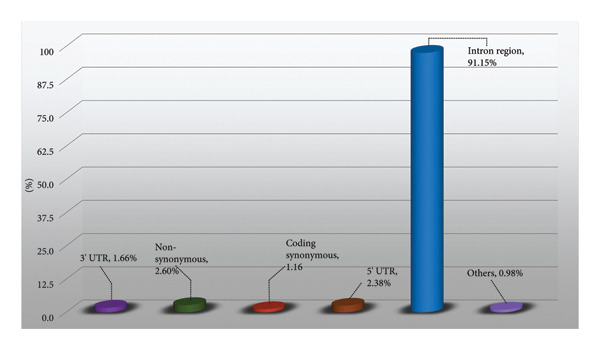
A graph depicting the prevalence of each type of SNP connected to the *ACADM* gene.

446 nsSNPs, 408 in 5′UTR, 284 in 3′UTR, 204 coding synonymous, 15,619 in intron sites, and the remaining (splice sites = 48, nonsense = 38, frameshift = 82) were found in the most comprehensive SNP database, dbSNP. 17,135 SNPs in total were extracted from this database. nsSNPs were selected from the whole roster. Furthermore, missense SNPs are validated using Ensembl. Ensembl helped us find 488 nsSNPs. Following the removal of duplicates, these nsSNPs are carefully evaluated. Figure 2 shows the SNPs that were retrieved.

### 3.2. Identified and Filtered Deleterious nsSNPs

**Figure 3:**
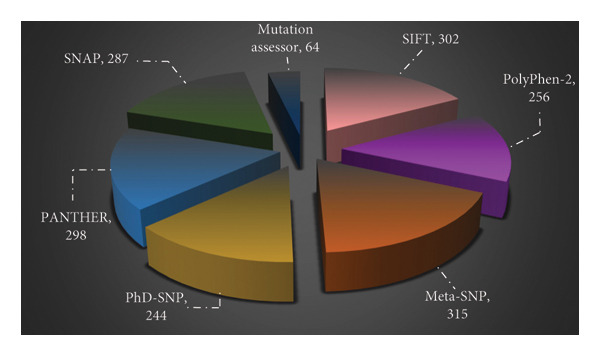
The proportion of SNPs that PANTHER, Meta‐SNP, PhD‐SNP, SNAP, PolyPhen‐2, SIFT, and MutationAssessor found to be potentially dangerous.

Seven distinct bioinformatics tools were applied to each of the nsSNPs in order to examine any probable physicochemical effects on the *ACADM* protein. The protein sequence of an understudied gene was retrieved from NCBI in the FASTA format (NCBI ID: NP_000007.1). As in silico technologies, MutationAssessor, PolyPhen‐2, SIFT, PhD‐SNP, SNAP, Panther, and Meta‐SNP were all used. Substitutions are characterized as “deleterious” with the score (TI = Total Index) of under 0.05 and as “tolerated” with the greater score, by SIFT [16, 17]. SIFT investigations revealed a connection between 302 nsSNPs and negative effects. Replacement‐specific damage is reflected via PolyPhen‐2, where the score near 1 demonstrates increased likelihood of harm [18]. PolyPhen‐2 suggests 256 nsSNPs as probably harmful. Likewise, the Meta‐SNP algorithm includes integration of statistical analysis, machine learning, or both to decide [19], which resulted in predicting 315 variations to be probably deleterious. To predict if a specific variation has the potential to alter protein form, PhD‐SNP employs a support vector machine, trained on features of raw data [20]. A thorough estimation indicated 244 variations as ill. Moreover, a novel algorithm is used by the PANTHER tool, which combines multiple sequence alignment, and the position‐dependent conservation calculation [21], resulting in the estimation of 298 variations as harmful. Furthermore, we applied the SNAP tool to group variations as functional or nonfunctional via the neural network‐based method [22]. It predicted that 287 disparities would be detrimental. Finally, the MutationAssessor tool was used to verify the variations [23]. MutationAssessor discovered 64 highly risky variations. A total of sixteen nsSNPs were selected (Figure 3), with negative predictions from all seven approaches.

### 3.3. MutPred Assessment for Structural and Functional Changes

MutPred was used to analyze the 16 nsSNPs that were chosen. The probability ratings for the returning findings are shown in Table 1. Among the anticipated consequences by the server were changes to the transmembrane protein, catalytic site, ordered interface, allosteric site, GPI‐anchor amidation, relative solvent accessibility, and metal binding. *p* values and statistics on previously established characteristics are shown in Table 1. Out of the 16 nsSNPs, only twelve were shown to change the protein function or structure at a threshold greater than 0.7. We went into further details regarding these 12 nsSNPs.

**Table 1:** MutPred 1.2 likelihood estimation of harmful variations, identified within the *ACADM* gene.

Mutations	*p* values
P62T	0.869
G99V	0.799
K142E	0.409
T193A	0.902
G195R	0.959
R206C	0.797
R206H	0.702
T228N	0.65
R243Q	0.604
C244R	0.963
S245L	0.919
G267R	0.934
R281T	0.902
V373A	0.907
I375T	0.855
R413C	0.678

### 3.4. *ACADM* Stability Assessment

The *ACADM* protein stability for 12 nsSNPs with residual changes was assessed using I‐Mutant. As shown in Table 2, all the variations were introduced individually, and stabilization was managed via RI scores (from 0 to 10). Every single nsSNP that was displayed had signs of declining stability. These results indicate that the 12 variations probably have a greater harmful influence on *ACADM* by decreasing stability, which makes more research necessary.

**Table 2:** I‐Mutant estimation of *ACADM* stability.

SNP id	Mutations	Stability
rs11549026	P62T	Decrease
rs370608001	G99V	Decrease
rs121434279	T193A	Decrease
rs121434278	G195R	Decrease
rs373715782	R206C	Decrease
rs200724875	R206H	Decrease
rs121434276	C244R	Decrease
rs121434281	S245L	Decrease
rs121434274	G267R	Decrease
rs121434282	R281T	Decrease
rs373057729	V373A	Decrease
rs121434275	I375T	Decrease

### 3.5. Evolutionary Conservation of *ACADM* Protein

Comprehending the evolutionary process is vital since variations may lead to health issues in humans [53]. The conservation trends of *ACADM* residues were examined via DeepREx‐WS to evaluate the probable consequences of the 12 chosen nsSNPs (Table 3). The positions of the 12 nsSNPs were found interesting, even though it provided per residue details within *ACADM*, where 44.42% of residues were exposed and 55.58% were buried. At 0.17 (conserved < 0.17; well conserved = 0.17), the conservation criterion was automatically determined. DeepREx‐WS only projected one residue, G99, to be highly functional, exposed, and conserved. T193, G195, R206, R281, V373, and I375 are anticipated to be among the other well‐preserved and subterranean structural relics. It was estimated that C244 and S245 would be buried residues with low conservation, whereas P62 and G267 would be exposed residues with poor conservation. After eliminating low‐conserved residues, eight highly conserved residues were chosen for more study. The findings demonstrated that all the variations found in highly conserved sites had the most detrimental influences on the form of *ACADM* protein.

**Table 3:** Evolutionary conservation trends of eight variations.

SNP id	Mutations	Conservation score	Prediction
rs11549026	P62T	0.14397	Low conservation and exposed
rs370608001	G99V	0.23534	High conservation and exposed
rs121434279	T193A	0.24021	High conservation and buried
rs121434278	G195R	0.20481	High conservation and buried
rs373715782	R206C	0.17612	High conservation and buried
rs200724875	R206H	1.17612	High conservation and buried
rs121434276	C244R	0.16385	Low conservation and buried
rs121434281	S245L	0.14147	Low conservation and buried
rs121434274	G267R	0.15006	Low conservation and exposed
rs121434282	R281T	0.17614	High conservation and buried
rs373057729	V373A	0.20903	High conservation and buried
rs121434275	I375T	0.21906	High conservation and buried

### 3.6. Structural Modeling of *ACADM* and Its Mutants

The eight most harmful nsSNPs (and the wild type) were screened to anticipate modifications in the *ACADM* protein and wild‐type sequence. 3D conformations of each mutant were then modeled via the Robetta server. Each *ACADM* variation was processed individually to create the 3D structures of mutant proteins. For each predicted structure, RMSD and TM scores were determined via TM‐align. The median difference between the α‐carbon backbones is measured via RMSD metrics, and the topological similarity is assessed using the TM score. Higher RMSD values demonstrated increased structural heterogeneity between structures. A subjective threshold of 1.25 Å was established. The two mutations with the greatest RMSD values, measuring 1.26 and 1.29 Å, respectively, were R206H (rs200724875) and R281T (rs121434282). The nsSNPs with the least variation were G99V (1.24 Å RMSD), G195R (1.22 Å RMSD), V373A (1.20 Å RMSD), T193A (1.19 Å RMSD), R206C (1.19 Å RMSD), and I375T (1.18 Å RMSD). Table 4 presents the RMSD values and TM scores. For remodeling via AlphaFold2, the two nsSNPs (R206H, R281T) with the highest RMSD values were chosen. The two mutations on top of the wild‐type *ACADM* protein are displayed in Figure 4, which was generated with PyMOL.

**Table 4:** TM and RMSD values of eight variations, estimated via TM‐align.

SNP Id	Mutation	TM score	RMSD
rs370608001	G99V	0.97365	1.24
rs121434279	T193A	0.97592	1.19
rs121434278	G195R	0.97398	1.22
rs373715782	R206C	0.97588	1.19
rs200724875	R206H	0.97361	1.26
rs121434282	R281T	0.97326	1.29
rs373057729	V373A	0.97568	1.2
rs121434275	I375T	0.97565	1.18

Figure 4(a) Wild‐type *ACADM*, together with its overlaid variant R206H, (b) wild‐type *ACADM*, together with its overlaid variant R281T, and (c) 3D model of the wild‐type *ACADM*.

**Figure:**
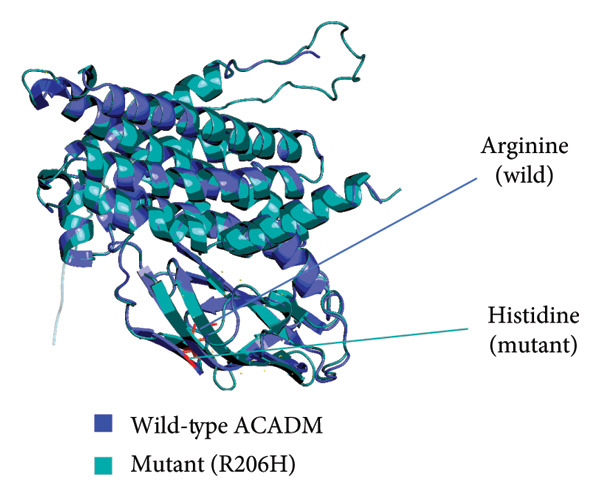
(a)

**Figure:**
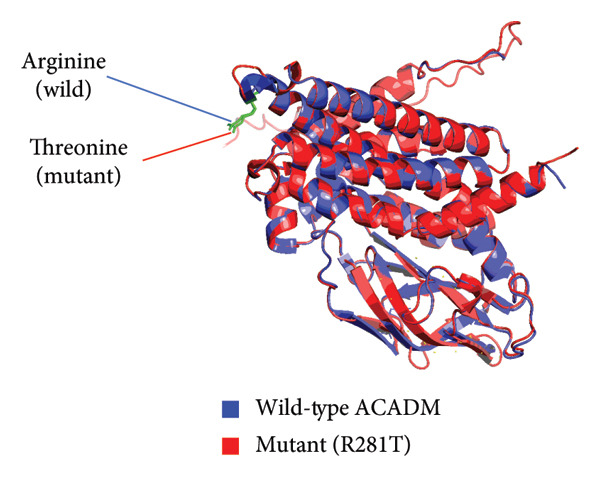
(b)

**Figure:**
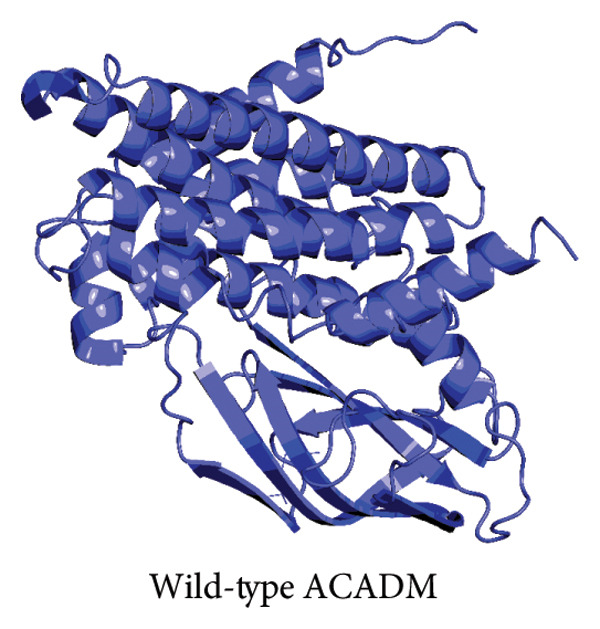
(c)

After prediction, MolProbity and the web‐based SAVES system were then used to evaluate these two models against the wild‐type *ACADM*. MolProbity discovered similar results for the wild‐type *ACADM* and the mutants. The greatest ERRAT scores were obtained by the mutant and wild‐type *ACADM* genes, with wild_type = 98.944, R206H = 98.941, and R281T = 98.944. A Ramachandran plot was generated for each predicted model to evaluate the stereochemical quality and backbone dihedral angle distribution of the wild‐type and mutant ACADM structures.

### 3.7. Molecular Docking Analysis

We docked *ACADM* mutants with the ETF protein to evaluate the binding potential and the interaction landscapes. We generated 10 distinct models for each complex (mutants and wild) via ClusPro, and one from each of them was chosen. With the maximum number of cluster members (94) and the lowest energy (−1341.4), the wild‐type–ETF complex stood out, indicating a stable and advantageous binding between them. Subsequently, the R206H–ETF complex displayed the binding potential (−1075.8) and the lowest cluster member (61), whereas the R281T–ETF complex displayed binding energy (−1080.1) with 93 cluster members. According to these findings (Table 5), R206H and R281T had the lower binding affinity with ETF and the greatest potential to interfere with the electron transfer mechanism, resulting in MCADD.

**Table 5:** Binding energies along with the cluster members of all complexes.

Protein	Binding energy	Cluster members
R206H	−1075.8	61
R281T	−1080.1	73
Wild type	−1341.4	94

A framework of interactions between the complexes was revealed through additional PDBsum analysis. Three interactions were used to evaluate the results: hydrogen bonds, which form intricate connections like small tunnels; salt bridges, which are electrostatic interactions, promoting the connections; and nonbonded contacts, which aid in the entire attraction trend. With 15 hydrogen bonds, 176 nonbonded contacts, and 1 salt bridge, the R281T mutant exhibited the fewest interactions with the ETF receptor. The R206H mutant formed 22 hydrogen bonds, 264 nonbonded contacts, and 4 salt bridges, demonstrating the fewest interactions (in comparison to the wild type). Finally, the strongest interactions were produced by wild‐type ACADM complexed with ETF, which formed 24 hydrogen bonds, 267 nonbonded contacts, and 5 salt bridges (Figure 5). According to these results, the R281T mutant interacts with ETF the least, followed by the mutant R206H and the wild type, potentially involving in the MCADD.

Figure 5(a) Wild‐type *ACADM*–ETF complex, along with their mutual interactions, (b) mutant R206H–ETF complex, along with their mutual interactions, and (c) mutant R281T–ETF complex, along with their mutual interactions.

**Figure:**
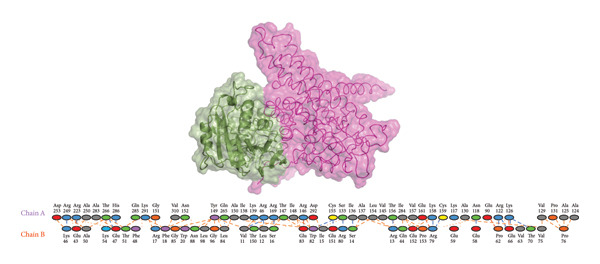
(a)

**Figure:**
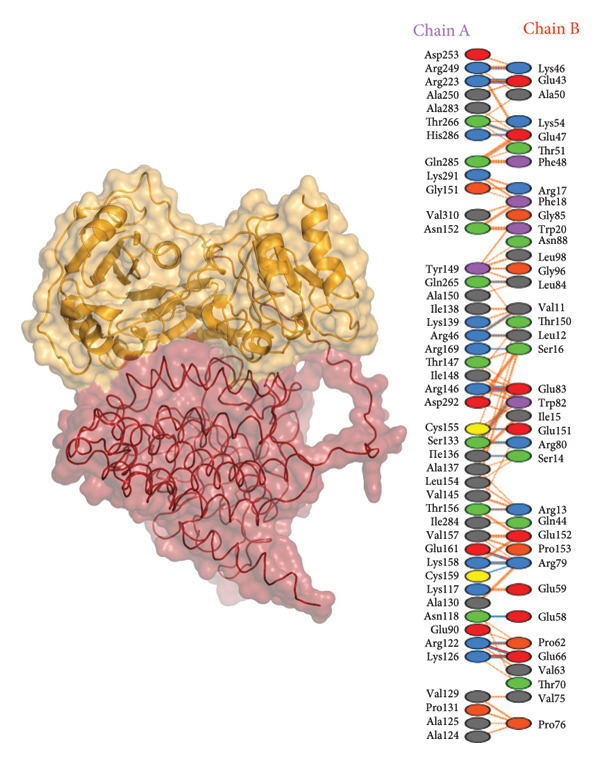
(b)

**Figure:**
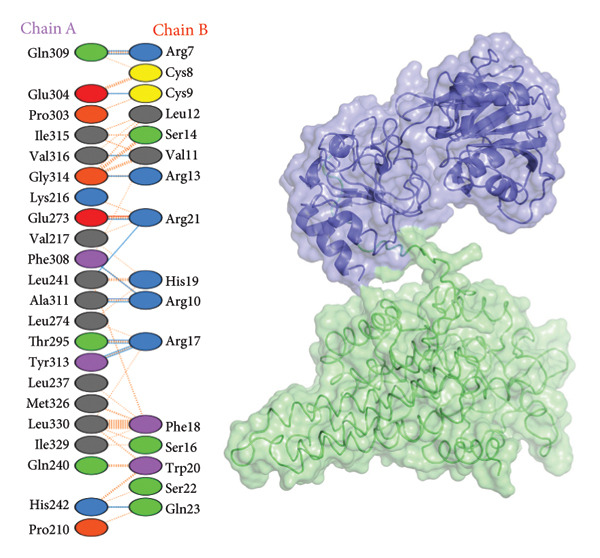
(c)

### 3.8. MD Simulation Analysis

The R206H and R281T variants showed fewer persistent interactions with the ETF (PDB ID: 1EFV) compared to the wild type. Via MD simulations, we investigated the time‐specific changing trend within these complexes over 200 ns. RMSD, root mean square fluctuation (RMSF), hydrogen bond analysis, and radius of gyration (*Rg*) were among the metrics used to evaluate the degree of structural changes and dynamic nature of the complexes. Throughout the simulation duration, these analyses shed light on the structural dynamics of these complexes. The results demonstrated considerable changes in dynamics and stability of both the mutants, together with their wild type, highlighting the significance of these variations on the overall complexes’ stabilities.

#### 3.8.1. RMSD Analysis

To reveal the therapeutic implications of the proposed variants, it is crucial to examine their binding trends via state‐of‐the‐art analyses. Their stability affects how much proteins interact and is crucial for the mechanistic exploration of deleterious mutants, allowing precise assessment of possible targets [54]. In this work, we computed the RMSD across time and analyzed modeled trajectories to investigate binding stability. We assessed and verified the stability of every complex, paying special attention to two chosen mutants, revealing variations in their stability. Over the course of the 200‐ns simulation, the wild‐type *ACADM*–ETF complex showed the highest stability, with an average RMSD between 3.8 and 4.2 Å, with a minor departure between 80 ns and 135 ns (Figure 6(a)). The R281T–ETF complex, on the other hand, showed the least stability; within the first 165 ns, its RMSD value increased to 17 Å before abruptly declining toward the end (Figure 6(b)). Similarly, over the course of the 200‐ns simulation, the RMSD of the L168Q–ETF complex increased to 7 Å, indicating persistent instability (Figure 6(c)). In general, the RMSD data suggested that both mutants showed increased instability relative to the wild type, likely altering their interactions with ETF, which ultimately leads to the MCADD.

Figure 6(a) RMSD of the wild‐type–ETF complex, (b) RMSD of the R281T–ETF complex, and (c) RMSD of the R206H–ETF complex.

**Figure:**
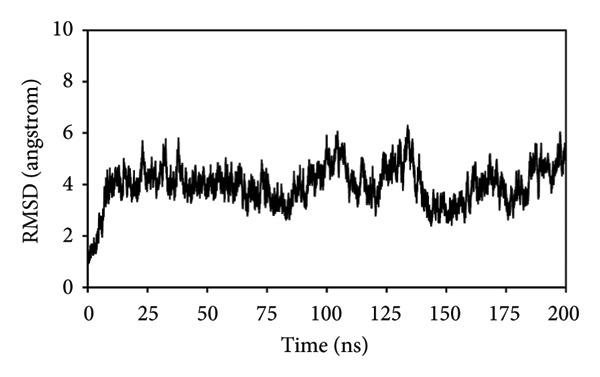
(a)

**Figure:**
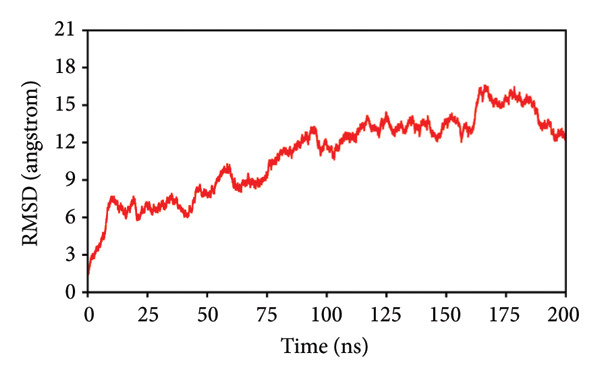
(b)

**Figure:**
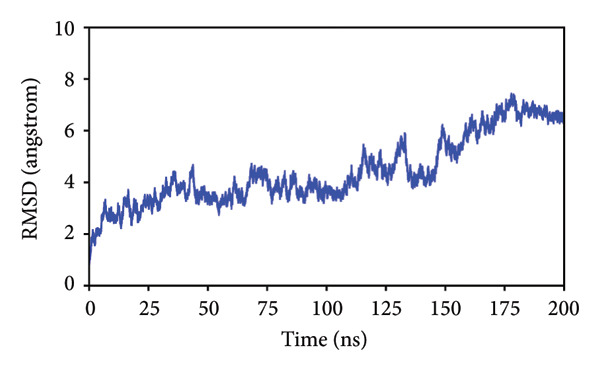
(c)

#### 3.8.2. RMSF Analysis

The RMSF analysis was performed on individual residues to evaluate the stability of the ETF active site during its interaction with the *ACADM* structure [55]. RMSF metrics were computed for the wild‐type *ACADM*–ETF complex and its R206H and R281T mutants, revealing distinct overall RMSF profiles for each modeled system. The *ACADM*–ETF complex demonstrated the lowest fluctuations, revealing the highest stability with RMSF values below 3 Å, except for some residues from 300–312, which showed fluctuations up to 6.7 Å (Figure 7(a)). Conversely, the R281T–ETF complex displayed the greater fluctuations, with an increase of up to 12 Å for the first 350 residues. The remaining residues fluctuated between 3 and 8 Å, indicating significant instability (Figure 7(b)). The R206H–ETF complex showed lower fluctuations relative to the R281T mutant but exhibited minimum stability than the *ACADM*–ETF complex. Overall, the residues in this complex showed minimal fluctuations under 3 Å, but some residues (i.e., 150–200, 315–350) exhibited the highest fluctuations, reaching up to 18 Å (Figure 7(c)). The RMSF data suggested that the residues within the *ACADM*–ETF complex maintained stable dynamics than those of the mutants over the 200‐ns simulation, suggesting that the R206H and R281T variations disrupted the *ACADM* interaction with ETF, potentially leading to MCADD.

Figure 7(a) RMSF of the wild‐type–ETF complex, (b) RMSF of the R281T–ETF complex, and (c) RMSF of the R206H–ETF complex.

**Figure:**
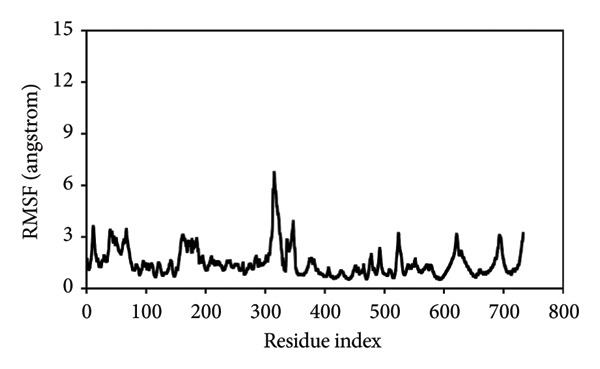
(a)

**Figure:**
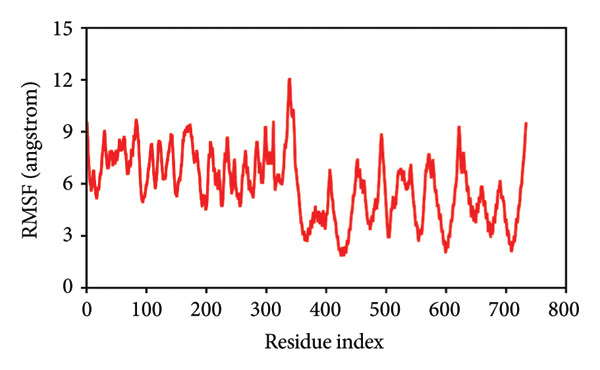
(b)

**Figure:**
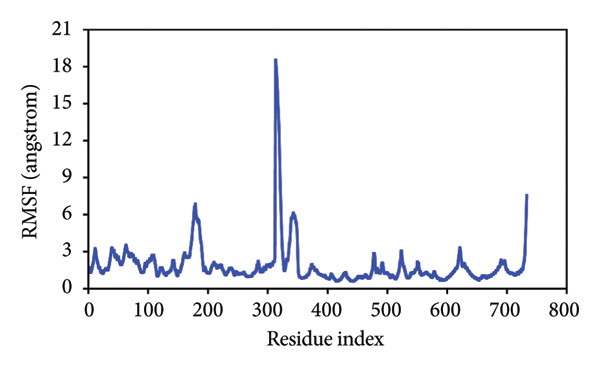
(c)

#### 3.8.3. *R* *g* Analysis

The structural compactness of each modeled complex was assessed by evaluating the *R* *g* as a time index to obtain insights into the dynamics and binding/unbinding events during the simulation [56]. The wild‐type *ACADM*–ETF complex had the maximum compactness of all complexes, as evidenced by its consistently low *R* *g* values, which remained under 37.2 Å (Figure 8(a)). This indicates a steady and structurally compact conformation of the *ACADM*–ETF complex. The R281T–ETF complex, on the other hand, showed the greater *R* *g* values, ranging from 52.3 to 52.5 Å, indicating the least structural compactness of the complexes (Figure 8(b)). This indicates a more extended and less compact structure for the R281T mutant complex. The R206H–ETF complex demonstrated similar behavior as the R281T–ETF complex, with *R* *g* values ranging from 47.3 to 47.4 Å throughout the simulation (Figure 8(c)), indicating a less compact structure relative to the wild type. The *R* *g* results corroborated the findings from the RMSD and RMSF analyses, further validating the higher stability and compactness of the wild‐type complex with the ETF protein compared to the R206H and R281T mutant complexes. The higher *R* *g* values for the mutant complexes suggest a more extended and flexible structure, potentially impacting the binding interactions with ETF and leading to reduced stability.

Figure 8(a) *R* *g* between the wild‐type–ETF complex, (b) *R* *g* between the R281T–ETF complex, and (c) *R* *g* between the R206H–ETF complex.

**Figure:**
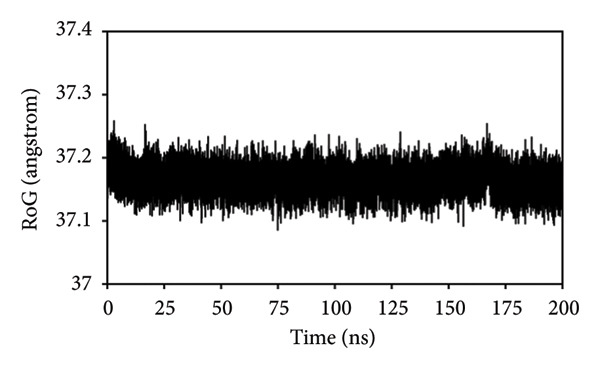
(a)

**Figure:**
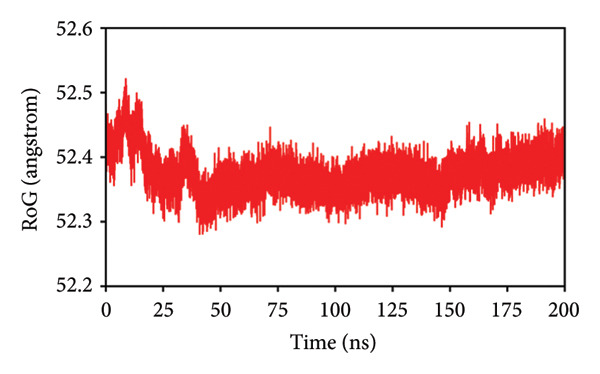
(b)

**Figure:**
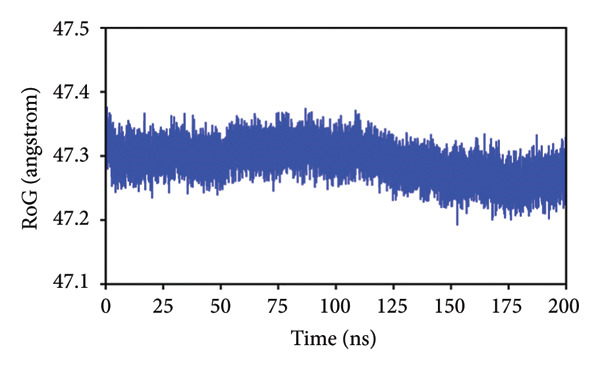
(c)

#### 3.8.4. Hydrogen Bond Analysis

Hydrogen and hydrophobic interactions at the contact point have a major impact on macromolecular interactions, particularly the interprotein bonds [57]. A maximum distance of 3.5 Å between the donor and acceptor atoms and an angle of 30 degrees between the hydrogen donor and acceptor were used to calculate the total number of hydrogen bonds established in each complex in order to assess the systems at the atomic level. These circumstances made it possible to accurately identify hydrogen bonds, which are essential for preserving the secondary structure of protein–protein complexes [58]. In comparison to the wild‐type *ACADM*–ETF complex, the R206H and R281T mutant complexes with ETF showed weaker hydrogen bonding networks, according to a time‐specific analysis. The mutant complexes showed a lower hydrogen bonds index relative to the *ACADM*–ETF complex (Figure 9). This implies that, in comparison to the wild‐type complex, the mutants’ attractive interaction with the ETF receptor is weaker. Ultimately, the reduced number of hydrogen bonds at the interface destabilized the mutant complexes, impacting their structural integrity and ability to form stable interactions with ETF, which is essential for proper MCAD function.

Figure 9(a) H‐bonding index between the wild‐type–ETF complex, (b) H‐bonding index between the R281T–ETF complex, and (c) H‐bonding index between the R206H–ETF complex.

**Figure:**
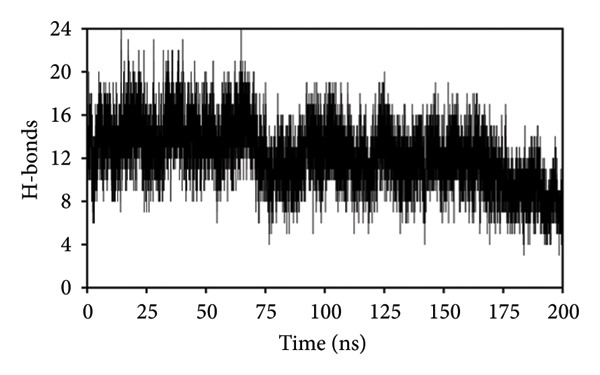
(a)

**Figure:**
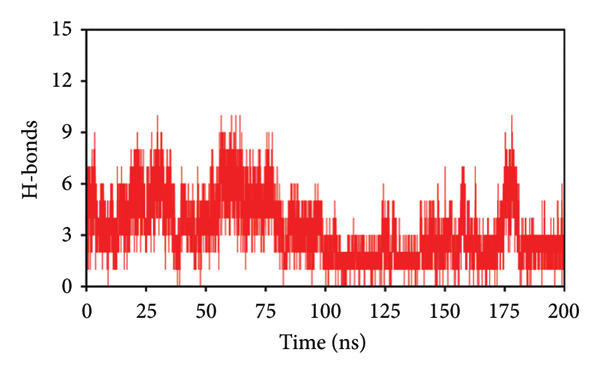
(b)

**Figure:**
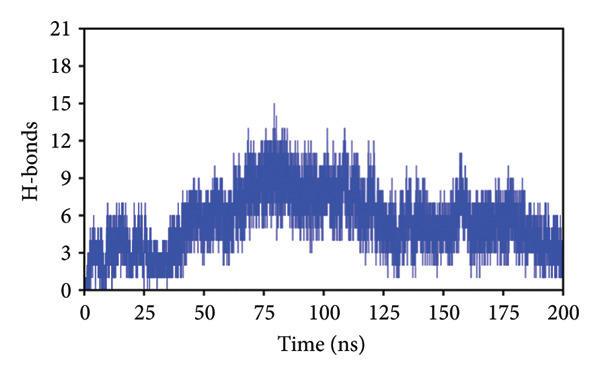
(c)

### 3.9. Predicted PTMs

#### 3.9.1. Methylation

No methylation of *ACADM* sites was projected by GPS‐MSP 3.0.

#### 3.9.2. Phosphorylation

GPS 6.0 and NetPhos 3.1 were used to predict the phosphorylation sites of *ACADM*, as shown in Figure 10. Thirty residues (Ser:13, Thr:10, and TyrL:07) have the potential to be phosphorylated, via NetPhos 3.1 projections. 24 residues (Ser:05, Thr:09, and Tyr:10) were revealed by GPS 6.0 as potentially phosphorylated.

Figure 10(a) Distribution of *ACADM* residues to be phosphorylated, (b) a graph showing residues in ordered and disordered regions, (c) phosphorylation graph of *ACADM* residues, obtained by NetPhos 3.1, (d) ubiquitination graph for *ACADM* residues, and (e) phosphorylation graph of *ACADM*, obtained by the GPS server.

**Figure:**
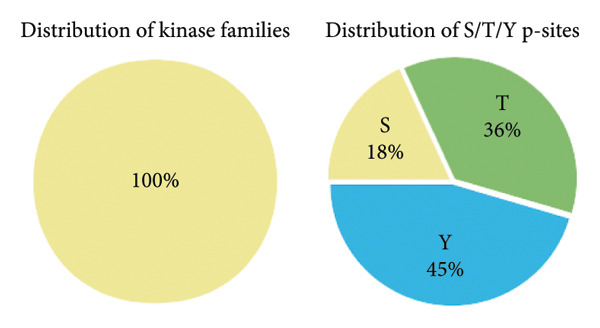
(a)

**Figure:**
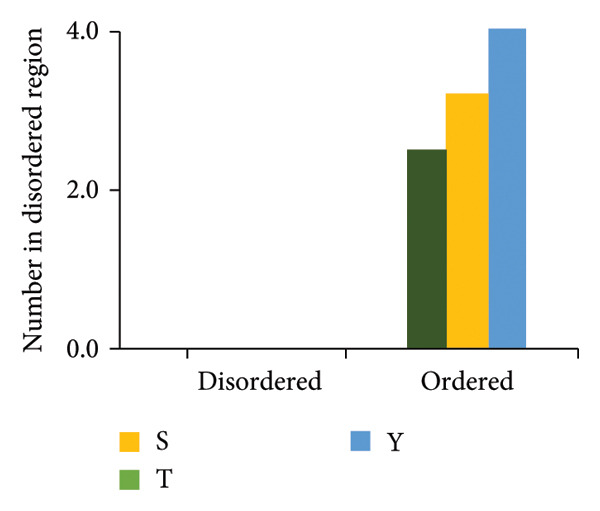
(b)

**Figure:**
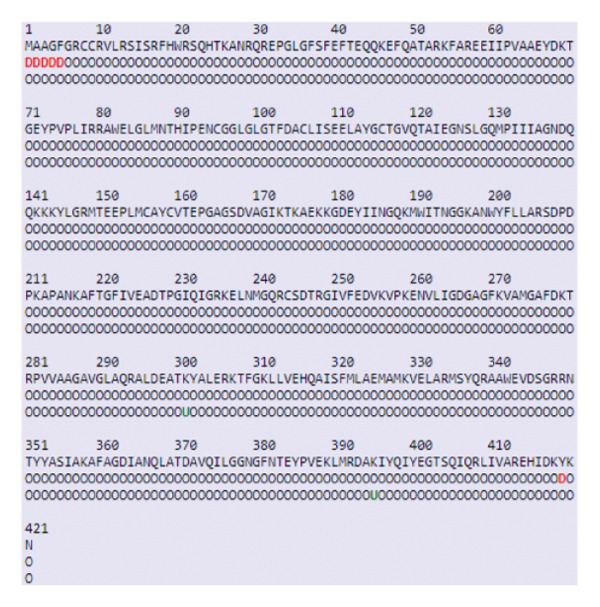
(c)

**Figure:**
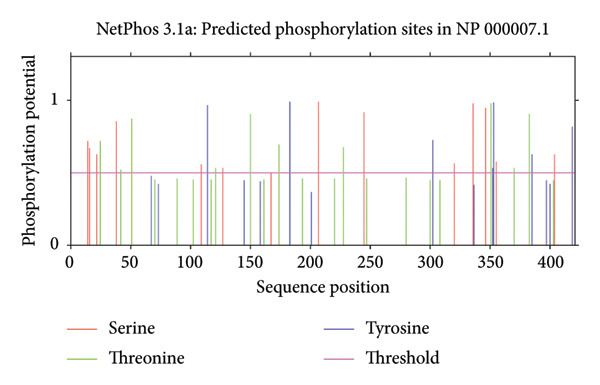
(d)

**Figure:**
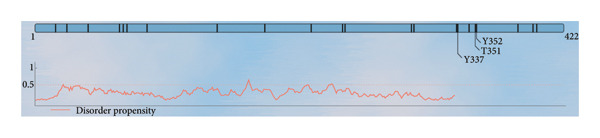
(e)

#### 3.9.3. Ubiquitination

The websites GPS‐Uber and RUBI were used to build the ubiquity prediction. Given their levels above the designated threshold (0.2655), we estimated using GPS‐Uber that 14 of 29 lysines at locations would be ubiquitinated. RUBI projected that two of the 29 lysine residues would be ubiquitinated. An exceptionally conserved or risky nsSNP area is projected to have no residues. It is estimated that 6.89% of all proteins are ubiquitinated.

#### 3.9.4. Glycosylation

To reveal the most likely glycosylation regions, NetOGlyc4.0 was utilized. Position 22, with a score of 0.92, was shown to be the site of glycosylation in the wild‐type *ACADM* protein. It is probable that this region is glycosylated.

### 3.10. *ACADM* Gene–Gene Interaction

*ACADM*’s connections with other genes were predicted using GeneMANIA and STRING. Many genes are predicted to physically interact with *ACADM*, such as *PPARA, ECHS1, CKAP5, HSD17B10, SDHC, ETFB, AASS,* and *STRAP*. In addition, *ACADM* is expressed along with *PPARA, ECHS1, CKAP5, ACADL, ACAD8, SDHA, SDHC, SDHD, ETFA, ETFB, ACADS, HADH, and DECR1*. In conjunction with *ACADM*, it is also expected that *GHITM, ACADS, ETFA, IVD,* and *HADH* would develop. *ACAD8, HDSA, HDSC, HDSD, GHITM*, and *HADH* are colocalized with it. Moreover, there are reciprocal genetic connections between *ACADM* and *AASS*. *ACADM*, *PPARA, CTNNA3*, and *FOXA2* go in the same direction. Furthermore, *ACADM, ACADS, ACAD8,* and *IVD* all share protein domains with one another. Each gene was given a total score based on STRING estimation. The GeneMANIA and STRING results are shown in Figure 11.

Figure 11Gene network assessed via GeneMANIA (a) and STRING (b).

**Figure:**
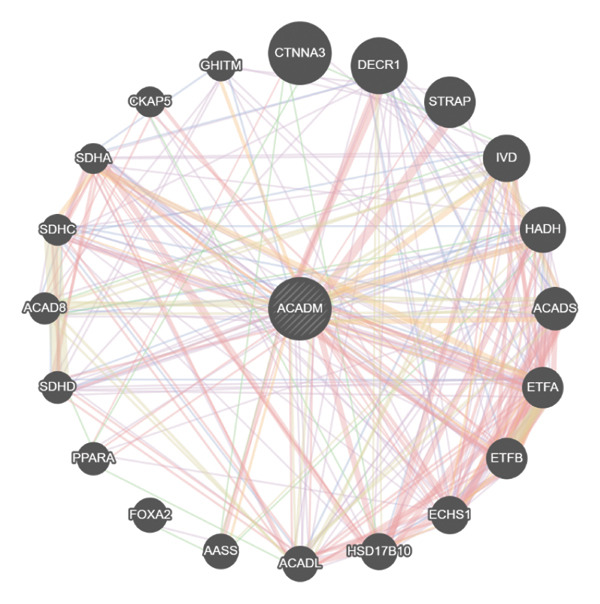
(a)

**Figure:**
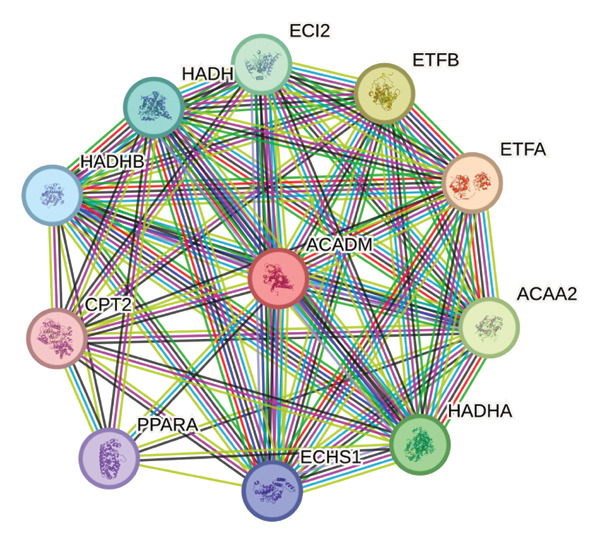
(b)

## 4. Discussion

An important genetic factor in determining an individual’s vulnerability for MCADD and the associated clinical consequences is the *ACADM* gene. Herein, we investigated how MCAD impairment is influenced by mutations in the *ACADM* gene. These results provide important data for further research and have a reasonable effect on how to understand the role of *ACADM* in MCADD, particularly in light of previous studies [11, 12]. It further suggests that an greater risk of MCADD is associated with nonfunctioning *ACADM* alleles. Strong evidence supporting the significance of *ACADM* gene mutations in MCADD is provided by our findings. Sixteen nsSNPs were shown to be harmful when a variety of analysis techniques were used. More research identifies the two most dangerous nsSNPs. Certain nsSNPs were deemed acceptable by some methods, such as PhD‐SNP and SIFT, but harmful by others. In order to ascertain if an amino acid is conserved, exposed, functional, or structural, DeepREx‐WS integrates evolutionary conservation data with predictions about solvent accessibility. It is believed that highly conserved residues have structural or functional significance since they are found on the protein’s surface and core [59]. Essential amino acids are probably more conserved since they are required for many biological functions, including interactions between proteins. Consequently, it has been discovered that protected regions have higher frequencies of the most harmful nsSNPs [60]. A single nsSNP, rs370608001, has been identified at position G99, a residue that is accessible, evolutionarily conserved, and structurally significant. Nonsynonymous SNPs were found at conserved, buried, and functionally significant residues. Examples of these SNPs are rs121434279 at position T193, rs121434278 at position G195, rs373715782 at position R206, rs121434282 at position R281, rs373057729 at position V373, and rs121434275 at position I375. It did not appear that the other nsSNPs in the *ACADM* protein had any structural or functional significance; they were either buried or exposed. All of the identified nsSNPs are predicted by the I‐Mutant web service to result in decreased protein stability. The internet web server Robetta served as the architectural model for both the selected nsSNPs and their wild type, *ACADM*. The resultant structures were adjusted using the RMSD data from TM‐align. Higher RMSD values (1.29) were found for nsSNPs containing rs200724875 at position R206H and rs121434282 at site R281T. The ERRAT’s Saves Server and MolProbity provide for further validation for these nsSNPs. Their Ramachandran plots in Procheck produced good results when they used input queries. Molecular docking with ETF protein, which facilitates the electron movement during dehydrogenation of medium‐chain acyl‐CoA molecules, validated the mutants as damaging. The results of MD simulation further demonstrated that the shortlisted nsSNPs caused the MCADD by disrupting the interaction with the ETF protein. Additionally, it has been demonstrated that PTMs influence the structures and functions of proteins and play a role in crucial biological processes such as cell signaling and protein–protein interactions [61, 62]. We investigated the potential impact of the chosen nsSNPs on the PTMs of the *ACADM* protein. We used a number of bioinformatics techniques to predict PTM sites in our poorly described proteins. Methylation is an important post‐translational alteration that affects gene expression because it changes how certain proteins interact with DNA. Another crucial protein control mechanism is the molecular switch, which allows a protein to perform functions including signal transduction pathways, protein activation and deactivation, and conformational changes in protein structure [63–66]. PPARA and *ACADM* are likely to interact the most, according to GeneMANIA and STRING. Alterations to the nsSNPs of the *ACADM* gene may impair the proper function of other genes involved, as indicated by the co‐expression profiles and interaction patterns. We considered all the relevant facts and data to identify the most harmful nsSNPs since our research was comprehensive. There are boundaries to any research, including ours. Mutations in the *ACADM* gene caused MCADD. These results suggest that *ACADM* gene changes may influence the development of various disorders as well as increase the risk of MCADD. Our findings align with previous studies that show an association between *ACADM* gene alterations and MCADD. The likelihood of MCADD is higher in those with malfunctioning *ACADM* alleles. Previous clinical studies have reported that rs121434282 (p.Arg281Thr) [67] variants in the ACADM gene are pathogenic and have been linked to MCADD. This mutation has been observed in affected individuals and are classified as deleterious in ClinVar (https://www.ncbi.nlm.nih.gov/clinvar/). Therefore, our findings are consistent with earlier reports indicating its potential role in disease development. It is important to note that there was not previous information reported on mutation rs200724875, indicating that further studies are needed for its validation. We specialize in statistical and mathematical approaches for computer programs and web servers. These results require confirmation through more experimentation. Our findings from multiple analyses of the gene’s nsSNPs including protein 3D structure, potential PTM sites, and gene–gene network may be useful in future research to further understand the relevance of the *ACADM* gene in enzyme deficiency. To reduce the burden of MCADD in individuals with *ACADM* gene mutations, more research is required to confirm our findings, develop novel therapeutic approaches, and identify causal relationships.

## 5. Conclusion

This study demonstrates that nsSNPs may affect the *ACADM* protein’s structure and functionality. Interestingly, two mutations of the native *ACADM* gene, R206H (rs200724875) and R281T (rs121434282), were found to be deleterious. Significant statistical evidence supports the significance of these nsSNPs in MCADD, as suggested by higher RMSD and MutPred scores. These SNPs significantly impact the pathophysiology of diseases associated with overexpression of *ACADM*, potentially advancing the field of precision medicine and therapy development. To determine how these polymorphisms affect the *ACADM* protein’s structure and function, more laboratory study is required. Analyzing *ACADM* mutations in various animal species may provide important information on the origins of the disease brought on by these genetic defects. Finally, our results welcome future investigations for a deeper comprehension and possible treatments by exposing the significance of certain *ACADM* mutations.

## Ethics Statement

The authors have nothing to report.

## Consent

The authors have nothing to report.

## Disclosure

The experimentation was conducted in accordance with applicable laws and ARRIVE guidelines.

## Conflicts of Interest

The authors declare no conflicts of interest.

## Author Contributions

Muhammad Waleed Iqbal and Muhammad Shahab: conceptualization, writing–original draft, reviewing, and editing. Yousef A. Bin Jardan and Mohammed Bourhia: formal analysis, investigations, funding acquisition, reviewing, and editing. Fakhreldeen Dabiellil: resources, funding acquisition, and supervision. Xinxiao Sun and Qipeng Yuan: validation and supervision.

## Funding

This work was financially supported by the Ongoing Research Funding Program (ORF‐2026‐457), King Saud University, Riyadh, Saudi Arabia.

## Data Availability

Data will be available upon request to the corresponding author.
